# Availability of Readable Online Spanish Rhinosinusitis Outcome Measures

**DOI:** 10.3390/jcm11247364

**Published:** 2022-12-12

**Authors:** Saangyoung E. Lee, Zainab Farzal, Adam J. Kimple, Ramón Moreno-Luna, Brent A. Senior, Brian D. Thorp, Charles S. Ebert

**Affiliations:** 1 Department of Otolaryngology, Head and Neck Surgery, University of North Carolina at Chapel Hill, Chapel Hill, NC 27599, USA; 2 Unidad de Rinología y Base de Cráneo, UGC de Otorrinolaringología, Hospital Universitario Virgen de la Macarena, 41009 Sevilla, Spain

**Keywords:** sinusitis, quality of life, patient reported outcome measure

## Abstract

Background: Patient-reported outcome measures (PROMs) are useful instruments that give providers insight into patients’ experiences with disease by quantifying the symptoms that matter most to patients. Results of these questionnaires can help guide management in chronic rhinosinusitis. However, these tools are often developed for native English speakers, which disadvantages others, who already have a language barrier to care. The aim of this study is to evaluate accessibility and readability of Spanish PROMs used to evaluate rhinosinusitis. Methods: Three Spanish readability measures, Gilliam, Peña & Mountain; SOL; and Fernandez-Huerta were used to evaluate PROMs utilized for rhinosinusitis. PROMs with sixth-grade readability level or easier were considered to meet health literacy recommendations. Results: Four Spanish PROMs utilized in assessment of rhinosinusitis were identified and evaluated. Cuestionario Español de Calidad de Vida en Rinitis (ESPRINT-15) was the most readable PROM and met readability recommendations in two of three measures. Nasal Obstruction Symptom Evaluation met suggested levels in one measure. The remainder of readability scores were more difficult than recommended. Conclusion: PROMs are powerful clinical tools that help patients communicate their symptoms and self-advocate. For providers to gain accurate and useful information, these measures should be written at appropriate readability levels. Most Spanish PROMs used for assessment of rhinosinusitis were above recommended readability. Development of future PROMs should ensure appropriate readability levels to provide good patient-centered care for our primarily Spanish speaking patients.

## 1. Introduction

Patient-reported outcome measures (PROMs) are standardized and validated questionnaires that convey a patient’s subjective disease experience to a healthcare team. The information that is gleaned can quantifying the extent of symptoms endured as well as elucidate the goals of care that matter most to patients. PROMs are widely used in the management of chronic rhinosinusitis (CRS), as medical decision making can be influenced by patients’ symptoms and subjective response to treatments [1]. Further, these surveys have been shown to improve symptom control, increase supportive care measures, and enhance overall patient satisfaction [2].

The utility of PROMs is inherently tied to a patient’s ability to understand and comprehend the questions asked to provide accurate responses describing their experiences. Studies have shown that the average American adult reads at an eighth-grade level, that of a typical 13–14 year old, and that health literacy is even lower [3,4,5,6]. With such widespread poor health literacy, the National Institutes of Health and other health organizations recommend that patient-targeted healthcare information be written at a sixth-grade reading level, that typical of a 11–12 year old, or lower [3,7,8,9]. There are many algorithms that have been developed to assess readability, which is defined as the comprehension level that a person must have to understand written materials, objectively measure difficulty of written material, and correlate to a grade level of education [10].

Readability of English PROMs utilized in chronic rhinosinusitis have been studied, with a majority having levels beyond those recommended [11]. However, PROMs for sinusitis written in Spanish have not been analyzed. 8% of Hispanic or Latino adults, the second largest demographic group in the United States, have been diagnosed with sinusitis, making the need for quality Spanish PROMs even more important [12]. The aim of this study is to evaluate accessibility and readability of Spanish PROMs used in rhinosinusitis.

## 2. Materials and Methods

### 2.1. Identification and Inclusion

This bibliometric review of PROMs was exempt from the Institutional Review Board (IRB). A total of seven Spanish PROMs used in the evaluation of rhinosinusitis were identified in the medical literature. PROMs that were not available in full text in the public domain were excluded. The excluded PROMs were the Polyposis Disability Index, Rhinitis Control Assessment Test, Rhinitis Control Assessment Test, Congestion quantifier, and Rhinoconjunctivitis Quality of Life Questionnaire [1,13,14,15].

The full text versions of the included PROMs were obtained by database search, manually converted to a text-based document, and then reviewed for accuracy by two of the co-authors, both Spanish speaking clinicians. Likert scale answer choices and answer choices with monosyllabic words were excluded from the readability assessment as these were deemed to have minimal cognitive burden on patients.

### 2.2. Readability Algorithms

Gilliam, Peña & Mountain (GPM) is a Spanish adaptation of the Fry readability formula, that compares sentences to syllables in a 100-word segment [16,17]. Readability, corresponding to a grade level, is calculated using the following algorithm:

$$\mathrm{GPM}=\left(3*\left(\frac{100}{s}\right)\right)/(\left(10+\frac{100}{s}\right)/t)$$

where s corresponds to the number of sentences in a 100-word sample, and t corresponds to the number of syllables in that sample.

SOL is a Spanish adaptation of the Simple Measure of Gobbledygook, which bases readability on the frequency of polysyllabic words in a text [18,19]. Readability, corresponding to a grade level, is calculated using the following algorithm:

$$\mathrm{SOL}=0.74*\left(1.043*\sqrt{P}+3.1291\right)-2.51$$

where P corresponds to the number of words with greater than three syllables in a ten-sentence sample.

Fernandez-Huerta (FH) is a Spanish adaptation of the Flesch Reading Ease readability formula, that compares sentences to syllables in a 100-word segment [20,21].

$$\mathrm{FH}=206.84-\left(0.6*t\right)-\left(1.02*s\right)$$

where s corresponds to the number of sentences in a 100-word sample, and t corresponds to the number of syllables in that sample. Readability grading scores are scaled as follows (Table 1):

## 3. Results

GPM, SOL, and FH average readabilities of included PROMs were 7.5, 9.8, and 70.0 respectively, which were all above the recommended sixth grade reading levels. ESPRINT-15 had the easiest readability with all three algorithms, meeting recommended levels in GPM and FH. NOSE-E met recommendation level with the FH algorithm only. The SOL algorithm consistently calculated the most difficult readability level for all PROMs. The SNOT-22, one of the most used PROMs in sinusitis, had among the most difficult readabilities across algorithms. See Table 2 for full details.

## 4. Discussion

PROMs are an important tool to that help patients to explain their personal experiences with their disease and are particularly helpful for providers in managing rhinosinusitis. Patient subjective scoring on these questionnaires can elucidate if treatment modalities are effective, or if other options including surgeries should be considered [22]. In chronic rhinosinusitis alone, based on a systematic review, there are fifteen commonly used PROMs [23]. In a striking comparison, there are seven Spanish PROMs used to evaluate any rhinosinusitis or sinonasal symptoms.

One shortcoming of our study is that three of the seven identified Spanish PROMs were not accessible in the public domain. However, included in the study is the SNOT-22, one of the most important questionnaires in CRS. It is utilized in evaluating quality of life in the core outcome set for trials of interventions in chronic rhinosinusitis (CHROME) [24,25]. This PROM has been shown to have strong internal consistency with great validity and reliability as a measurement tool in CRS; however, it has a greater difficulty of readability against other PROMs analyzed. Though the readability is more difficult, the SNOT-22 elucidates a wider breadth of information on patient experiences in CRS than other PROMs, with nasal-related, facial-related, quality-of-life, and psychologic questions.

Based on our analysis, it is evident that PROMs used to evaluate rhinosinusitis symptoms for native Spanish speakers are often not written at appropriate readability levels. The most appropriate questionnaire was the ESPRINT-15, which was originally written for Spanish speakers, compared to the rest of the analyzed PROMs, which were translated from questionnaires originally written for native English speakers. Interestingly, a study looking at readability comparisons between original texts and translated patient information leaflets showed generally more difficult readability with original texts [26]. This was attributed to the nature of the Spanish language that has longer and syntactically more complex sentence than an English sentence [26]. With the readability algorithms utilizing words per sentence as a measure of readability difficulty, a straight English to Spanish translation would generally be calculated to have easier readability. This could potentially incur loss of fidelity in translations of longer passages. However, in PROMs, which utilize individual questions, this would be less concerning.

Health literacy is critically important to allow patients to understand and participate in their care, and to help them make informed decisions [27]. Lower health literacy is directly correlated to barriers to care and poorer health outcomes [28]. A 2003 national literacy assessment illustrated that 35% of adults without basic health literacy identified as Hispanic [4]. Hindered already by socioeconomic factors such as health literacy, native Spanish speakers further are marginalized with a language communication with their healthcare providers. This language gap can lead to poor and adverse outcomes that lead to a disparity in care compared to English speaking patients [29,30]. With this in mind, it is critical to bridge communication and language barriers with more easily readable PROMs to provide quality patient-centric care.

## Figures and Tables

**Table 1:** FH readability grading scale.

FH Score	Readability Difficulty	Grade Level
0–30	Very difficult	University
30–50	Hard	Selective Courses
50–60	Somewhat hard	Pre-University
60–70	Normal	7–8
70–80	Somewhat easy	6
80–90	Easy	5
90–100	Very easy	4

**Table 2:** Readability scores for Spanish PROMs used in management of rhinosinusitis. Recommended readability levels are at a sixth-grade level or easier for GPM and SOL, and 60–70 or greater for FH.

	Gilliam, Peña & Mountain (GPM) (Reading Grade Level)	SOL (Reading Grade Level)	Fernandez-Huerta (FH) (Standardized Score)
Nasal Obstruction Symptom Evaluation (NOSE-E)	8	9.5	70.7
Rhinitis Control Assessment Test (RCAT)	8	11.6	69.5
Cuestionario Español de Calidad de Vida en Rinitis (ESPRINT-15)	6	8.3	74.7
Sino-nasal Outcome Test (SNOT-22)	8	9.8	65.2
Average	7.5	9.8	70.0

## Data Availability

Not applicable.
